# Impact of preanalytical sample handling on GLP-1 measurement in porcine plasma: A pilot ELISA-based study

**DOI:** 10.17221/34/2026-VETMED

**Published:** 2026-10-09

**Authors:** Lubomir Culka, Jaroslav Novotny, Lenka Leskova, Zuzana Krepelkova, Viera Karaffova, Michaela Madarova, Petra Schusterova

**Affiliations:** ^1^Clinic for Swine, University Veterinary Hospital, University of Veterinary Medicine and Pharmacy in Kosice, Kosice, Slovak Republic; ^2^Department of Public Veterinary Medicine and Animal Welfare, University of Veterinary Medicine and Pharmacy in Kosice, Kosice, Slovak Republic; ^3^Department of Morphological Disciplines, University of Veterinary Medicine and Pharmacy in Kosice, Kosice, Slovak Republic; ^4^Clinic for Ruminants, University Veterinary Hospital, University of Veterinary Medicine and Pharmacy in Kosice, Kosice, Slovak Republic; ^5^Department of Microbiology and Immunology, University of Veterinary Medicine and Pharmacy in Kosice, Kosice, Slovak Republic

**Keywords:** DPP-4 inhibitor, ELISA, GLP-1, porcine plasma, sample handling, sample stability

## Abstract

Glucagon-like peptide-1 (GLP-1) is an incretin hormone that plays a key role in the regulation of glucose metabolism; however, it is highly sensitive to biological sample handling conditions. The aim of this pilot study, conducted in two piglets and two adult finishing pigs, was to compare the effects of selected preanalytical factors on measured GLP-1 concentrations in porcine plasma and to identify conditions suitable for its reliable determination using ELISA. The evaluated factors included blood collection tube type (EDTA vs EDTA with a DPP-4 inhibitor), centrifugation temperature (4 °C vs room temperature), and sample processing conditions (fresh vs frozen samples). In addition, the influence of the physiological status of the animals (fasting vs post-glucose administration) was assessed. Samples collected in tubes containing a DPP-4 inhibitor showed higher measured GLP-1 concentrations than standard EDTA samples. Samples processed at room temperature showed higher GLP-1 concentrations compared to samples centrifuged at 4 °C. The obtained results suggest that sample handling conditions may significantly influence GLP-1 determination by ELISA. The use of EDTA tubes containing a DPP-4 inhibitor combined with rapid sample processing and centrifugation at 4 °C may represent a suitable procedure for standardised GLP-1 measurement.

Glucagon-like peptide-1 (GLP-1) and glucose-dependent insulinotropic polypeptide (GIP) are among the principal representatives of the incretin hormone family. They are released from the intestine after food intake and play an important role in regulating glucose homeostasis (Gautier et al. 2005). Their primary effect is to reduce blood glucose concentration by stimulating insulin secretion and suppressing glucagon secretion; this effect depends on elevated blood glucose levels (i.e., it is glucose-dependent). In addition to its pancreatic effects, GLP-1 slows gastric emptying and reduces food intake through its action on the central nervous system (Holst 2007). These pleiotropic effects make GLP-1 an important target for pharmacological research in treating obesity and type 2 *diabetes mellitus* (Nauck and Meier 2018).

Under physiological conditions, GLP-1 is rapidly metabolised, mainly by the enzyme dipeptidyl peptidase-4 (DPP-4) (Deacon 2004; Baggio and Drucker 2007). Cleavage of the *N*-terminal end results in the formation of the inactive metabolite GLP-1(9–36). This enzymatic inactivation represents the principal mechanism regulating its biological activity and ensures the short duration of hormone action, thereby contributing to the prevention of hypoglycaemia (Drucker 2006).

GLP-1 is a natural substrate of DPP-4; therefore, inhibiting this enzyme is a therapeutic strategy to increase active GLP-1 levels and enhance its antihyperglycaemic effects (Deacon et al. 1995). Consequently, modulation of the GLP-1/DPP-4 axis is widely used in clinical practice and pharmacological research for the treatment of metabolic disorders, particularly *diabetes mellitus* (Albrechtsen et al. 2014).

Due to the high lability of GLP-1, minimising its degradation during the preanalytical phase is essential for standardised determination, particularly through appropriate blood collection, sample stabilisation, and plasma storage procedures. The aim of this study was to compare different processing conditions of porcine blood samples and to identify the most suitable procedure for the stabilisation and standardised determination of GLP-1 in plasma using ELISA.

## MATERIAL AND METHODS

The study consisted of two experimental parts. The first part included postnatal monitoring of crossbred piglets (Pietrain × Duroc × Landrace; *n* = 2; females) during the first four weeks of life, whereas the second part included two adult finishing pigs (*n* = 2; females) used to evaluate the effects of selected preanalytical factors on GLP-1 determination. Blood sampling in piglets was performed at different developmental stages (T0–T5; Table 1). Piglets were weaned at 28 days of age (T4), and the final sampling point (T5) was performed three days after weaning.

**Table 1:** Sampling schedule for piglets monitored during the study (T0–T5)

Time point	Age/physiological stage
T0	before first colostrum intake
T1	day 7 of life
T2	day 14 of life
T3	day 21 of life
T4	weaning day
T5	3 days after weaning

From 7 days of age, piglets were offered a commercial creep feed (ENERGYS Weaning Pellets; De Heus s.r.o., Kendice, Slovak Republic). Following weaning, they were fed a commercial starter diet (ENERGYS A1 Start GR; De Heus s.r.o., Kendice, Slovak Republic). Animals were housed at the Clinic of Swine, University of Veterinary Medicine and Pharmacy in Košice, Slovakia, under standard indoor husbandry conditions with free access to water. Piglets remained with the sow until weaning at 28 days of age, whereas adult finishing pigs were housed in conventional indoor pens. No experimental treatment groups were established, and the same animals were monitored repeatedly throughout the study period. Therefore, randomisation was not applied. In piglets, blood was collected from the orbital sinus (*sinus ophthalmicus*), whereas in adult pigs, sampling was performed from the jugular vein (*vena jugularis*). Adult pigs were fasted for 12 h before the first blood collection. The first blood sample was obtained under fasting conditions. Subsequently, 60 g of glucose powder was administered orally by placing it into the feed trough. The glucose was consumed completely within approximately 1 minute, and the second blood sample was collected approximately 30 min later.

No analgesia was administered because blood sampling was performed as a routine minimally invasive veterinary procedure by experienced veterinary personnel in accordance with the approved ethical protocol. Blood was collected into two types of tubes. The first type consisted of standard EDTA tubes used as the standard anticoagulation condition. The second type consisted of commercial BD P800 tubes (BD, Franklin Lakes, NJ, USA) containing EDTA together with protease inhibitors, including a dipeptidyl peptidase-4 (DPP-4) inhibitor, designed for stabilisation of peptide hormones during the preanalytical phase. The collected blood volume ranged from approximately 2 ml in piglets to 4 ml in adult pigs.

Immediately after collection (approximately within 5 min), samples were processed by centrifugation for 15 min at 1 000 × *g.* Two centrifuges were used simultaneously: a refrigerated centrifuge (4 °C) and a standard laboratory centrifuge (room temperature). Samples assigned to 4 °C were placed directly into the precooled centrifuge, whereas samples assigned to room temperature were centrifuged simultaneously under identical conditions to ensure that centrifugation temperature represented the only experimental variable. After plasma separation, samples were either analysed immediately on the same day or frozen at –80 °C and stored for a maximum of 2 months before analysis. For the comparison of fresh and frozen plasma, plasma obtained from the same blood samples was first analysed on the day of collection, then stored at –80 °C, thawed once after storage, and reanalysed using the same ELISA kit in a separate analytical run.

The study protocol was reviewed and approved by the Ethics Committee of the University of Veterinary Medicine and Pharmacy in Košice, Slovakia (Approval No. EKVP/2026-4). All procedures involving animals were conducted in accordance with Directive 2010/63/EU of the European Parliament and of the Council on the protection of animals used for scientific purposes, the applicable national legislation of the Slovak Republic, and the institutional guidelines for the care and use of animals.

GLP-1 concentrations were determined using a commercial Pig Glucagon Like Peptide-1 (GLP-1) ELISA Kit (Cusabio Biotech Co., Ltd., Wuhan, P.R. China). The kit enabled determination of total GLP-1, including both active and degraded forms of the hormone. Analyses were performed according to the manufacturer’s protocol, and each sample was measured in duplicate; the final value represented the mean of the two measurements. Optical density was measured at a wavelength of 450 nm using a Revelation instrument (DYNEX Technologies, Chantilly, VA, USA) with Revelation QuickLink v4.25 software (DYNEX Technologies, Chantilly, VA, USA). GLP-1 concentrations were determined by interpolation from a standard curve prepared from serial dilutions of the standard supplied with the kit. According to the manufacturer, the analytical sensitivity (minimum detectable dose) of the assay was <3.9 pg/ml, and the detection range was 15.6–1 000 pg/ml. According to the manufacturer’s validation data, the assay demonstrated an intra-assay coefficient of variation (CV) of <8% and an inter-assay CV of <10%. Because all samples could not be analysed simultaneously, the 96-well break-apart microplate supplied with the kit was used in two analytical runs according to the manufacturer’s instructions. Unused strips were stored according to the manufacturer’s recommendations and used for the second analytical run. Each analytical run included its own standard curve, and all plasma samples were analysed in duplicate using the same analytical protocol. The experimental design of the study, including all evaluated preanalytical factors and biological groups, is summarised in Table 2.

**Table 2:** Experimental design

Comparison	Animals	Variables	Purpose
Developmental monitoring	2 piglets	T0–T5	physiological changes
Tube type	2 adult pigs	EDTA vs BD P800	DPP-4 inhibition
Centrifugation	2 adult pigs	4 °C vs RT	processing temperature
Storage	2 adult pigs	fresh vs frozen	sample stability
Physiological status	2 adult pigs	fasting vs glucose	physiological comparison

BD P800 = blood collection tube containing EDTA and protease inhibitors; DPP-4 = dipeptidyl peptidase-4; EDTA = ethylenediaminetetraacetic acid; RT = room temperature

The overall experimental workflow and sample allocation are illustrated in Figure 1. Overall, 28 plasma samples were analysed within the study.

**Figure 1:**
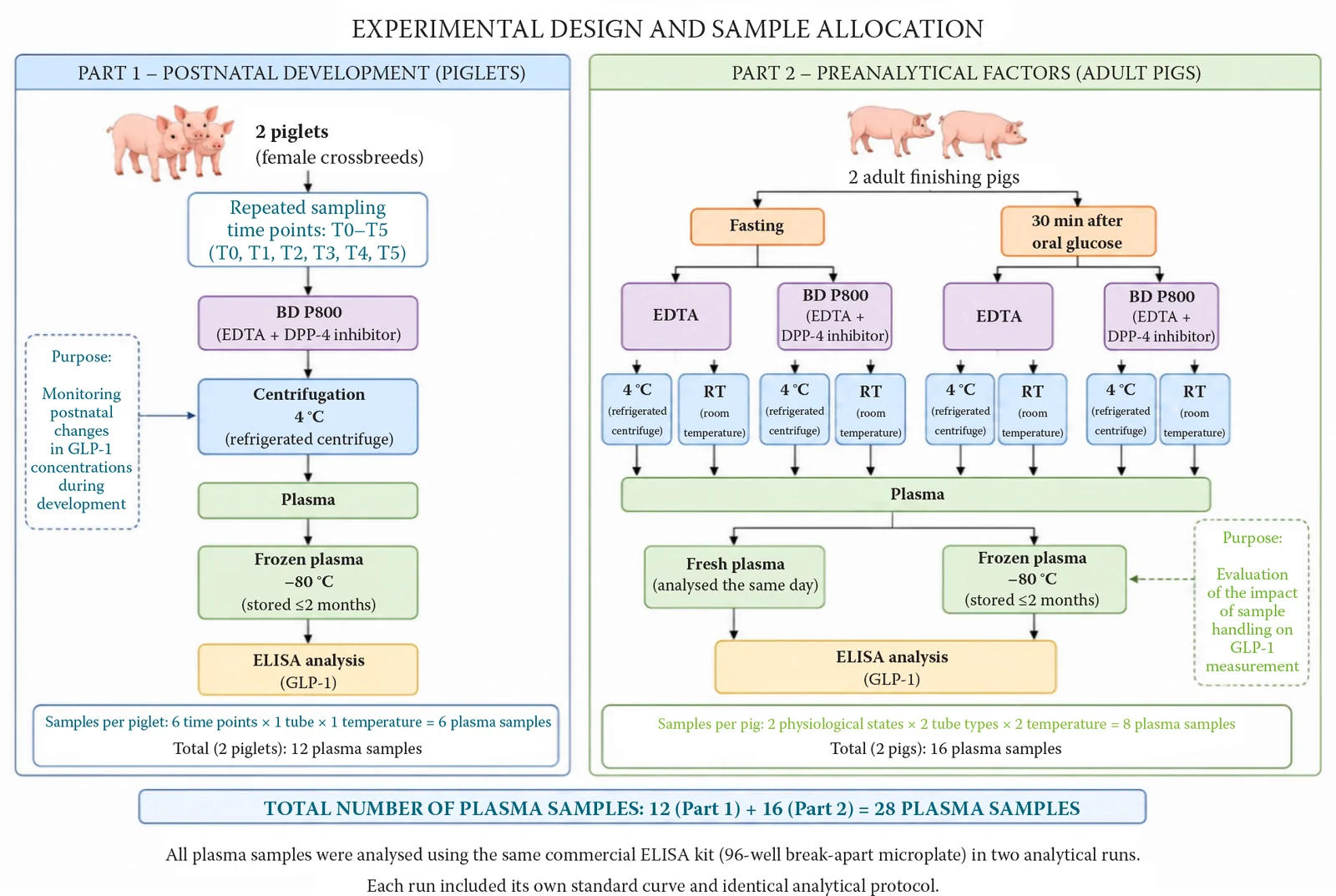
Schematic overview of the experimental design and sample allocation used in the study Part 1 illustrates the longitudinal monitoring of GLP-1 concentrations in two female piglets during the postnatal period (T0–T5). Part 2 illustrates the evaluation of selected preanalytical factors affecting GLP-1 determination in two adult female pigs under fasting and post-glucose conditions BD P800 = blood collection tube containing EDTA and protease inhibitors; DPP-4 = dipeptidyl peptidase-4; EDTA = ethylenediaminetetraacetic acid; GLP-1 = glucagon-like peptide-1; RT = room temperature; T0–T5 = sampling time points during postnatal monitoring

## RESULTS

The effects of preanalytical sample handling on measured GLP-1 concentrations are summarised in Table 3. In both adult pigs, GLP-1 concentrations measured 30 min after glucose administration were higher than those measured during fasting. Centrifugation temperature also influenced the measured GLP-1 concentrations, with samples centrifuged at room temperature consistently showing higher values than samples processed at 4 °C under both fasting and post-glucose conditions. Similarly, blood samples collected into BD P800 tubes containing a DPP-4 inhibitor showed higher GLP-1 concentrations than samples collected into standard EDTA tubes in both animals. Differences were also observed between fresh and frozen plasma samples (Table 3). Under the experimental conditions of this study, the highest GLP-1 concentrations were observed in fresh plasma collected after glucose administration into BD P800 tubes, which represented the reference condition for comparison of the evaluated preanalytical factors.

**Table 3:** Individual GLP-1 concentrations measured under different experimental conditions

Factor	Condition	Animal 1 (pg/ml)	Animal 2 (pg/ml)
Physiological status	fasting	37.45	99.90
	after glucose	79.95	116.95
			
Centrifugation temperature	4 °C (fasting)	33.70	41.20
	room temperature (fasting)	87.50	112.30
	4 °C (after glucose)	78.30	81.60
	room temperature (after glucose)	103.70	130.20
			
Blood collection tube	standard EDTA	25.50	82.50
	BD P800	79.95	116.95
			
Sample storage	fresh plasma	79.95	116.95
	frozen plasma	84.20	91.15

Each value represents the mean of duplicate ELISA measurements obtained from one adult finishing pig

BD P800 = blood collection tube containing EDTA and protease inhibitors; EDTA = ethylenediaminetetraacetic acid; GLP-1 = glucagon-like peptide-1

In piglets, repeated measurements demonstrated considerable inter-individual variability in GLP-1 concentrations throughout the monitored postnatal period (Figure 2). Both animals showed low GLP-1 concentrations at T0 (before first colostrum intake), followed by an increase after the initiation of enteral feeding. Thereafter, the temporal pattern differed between the two piglets. Piglet 2 maintained consistently higher GLP-1 concentrations than Piglet 1 at all subsequent sampling points, reaching the highest value at T3, whereas Piglet 1 showed more moderate fluctuations during the monitoring period. Owing to the limited number of animals, these findings should be regarded as descriptive observations only.

**Figure 2:**
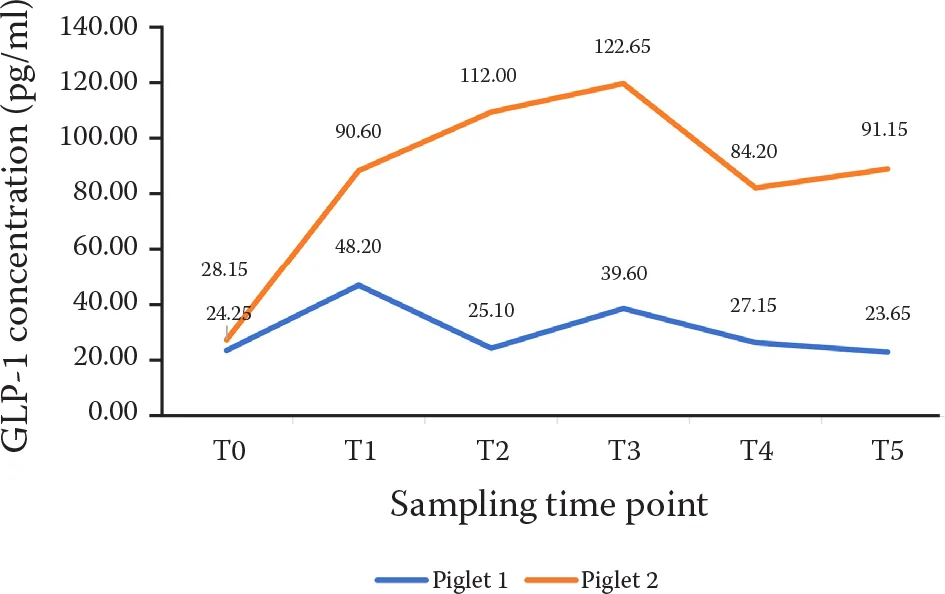
Individual GLP-1 concentrations in two piglets during the postnatal period (T0–T5). Values represent the mean of duplicate ELISA measurements GLP-1 = glucagon-like peptide-1; T0–T5 = sampling time points during postnatal monitoring

## DISCUSSION

This study evaluated the effects of different sample-handling conditions on GLP-1 determination in porcine plasma using ELISA. The present pilot study suggests that sample manipulation procedures may significantly influence the measured concentrations of the analysed hormone.

Higher GLP-1 concentrations after glucose administration than in fasting samples reflect the well-known physiological effect of the incretin system, in which nutrient intake stimulates GLP-1 secretion from enteroendocrine L-cells (Baggio and Drucker 2007). This finding supports the assumption that the applied methodology could detect physiological changes in GLP-1 concentrations.

Preliminary monitoring of GLP-1 concentrations in piglets indicated possible changes associated with postnatal development and the introduction of enteral nutrition. Mean GLP-1 concentrations immediately after birth (T0) were lower than those recorded at later sampling time points. Knapper et al. (1995) reported similar observations, showing increased plasma GLP-1 concentrations after milk intake in piglets. However, because repeated measurements were performed in only two animals, these findings should be considered preliminary and cannot be used to draw general conclusions regarding the development of the incretin system in pigs.

An important finding of this study was the difference between samples collected into standard EDTA tubes and BD P800 tubes containing a DPP-4 inhibitor. Higher GLP-1 concentrations in samples stabilised with a DPP-4 inhibitor suggest that the use of EDTA alone may not be sufficient to preserve GLP-1 stability during sample processing. This finding is consistent with previous reports describing rapid GLP-1 degradation by the DPP-4 enzyme (Deacon et al. 1995; Deacon 2004), as well as with the observations of Yi et al. (2015), who demonstrated improved GLP-1 stability in P800 tubes.

We observed a numerical difference between samples centrifuged at 4 °C and those processed at room temperature. Samples centrifuged at room temperature showed higher GLP-1 concentrations, even when tubes containing a DPP-4 inhibitor were used. Since the applied ELISA kit measured total GLP-1, including both active and degraded hormone forms, these differences are unlikely to be explained solely by enzymatic GLP-1 degradation. The findings rather suggest a potential influence of plasma matrix alterations on immunochemical determination (Chiu et al. 2010). One possible explanation is haemolysis or platelet activation during sample processing at higher temperatures, which may result in the release of intracellular components and subsequent matrix effects interfering with ELISA analysis. Similar interferences affecting immunochemical methods have also been described in other studies (Tate and Ward 2004; Saracevic et al. 2019). Soderstrom et al. (2016) further demonstrated that centrifugation conditions may influence platelet activation and plasma composition.

Immunochemical methods may be more sensitive to matrix effects than analytical techniques based on mass spectrometry, such as MALDI-TOF MS (Yi et al. 2011). This difference may partially explain why Yi et al. (2015), when analysing stabilised samples, did not observe a significant negative effect of room-temperature centrifugation, whereas in the present study differences between cooled and non-cooled samples were observed during ELISA determination.

The results of this study suggest that not only DPP-4 inhibition, but also strict control of sample handling conditions, including centrifugation temperature, is important for reliable GLP-1 determination. From a practical point of view, the use of tubes containing a DPP-4 inhibitor together with rapid sample processing and centrifugation at 4 °C remains the recommended procedure according to current guidelines for peptide stabilisation, despite the higher GLP-1 concentrations observed after room-temperature centrifugation in the present study. This study is limited by the low number of animals, the absence of haemolysis assessment, and the fact that individual feed intake of piglets was not monitored, which prevents exclusion of its potential influence on GLP-1 concentrations. These limitations prevent unequivocal interpretation of the observed differences. Future studies should include a larger sample set, haemolysis control, monitoring of individual feed intake, and analysis of additional enzymes involved in GLP-1 degradation, such as NEP 24.11 (Plamboeck et al. 2005).

Sample handling conditions may substantially influence measured GLP-1 concentrations in porcine plasma determined by ELISA. Within this pilot study, the use of blood collection tubes containing a DPP-4 inhibitor combined with rapid sample processing and centrifugation at 4 °C should be considered the recommended procedure for standardised GLP-1 determination, based on current knowledge of GLP-1 stabilisation. Differences in measured GLP-1 concentrations were also observed depending on centrifugation temperature, suggesting that preanalytical conditions may influence immunochemical GLP-1 determination. Because the study was performed on a limited number of animals, these findings should be considered preliminary methodological observations requiring confirmation in a larger sample set. From a practical perspective, use blood collection tubes containing a DPP-4 inhibitor, process samples rapidly, centrifuge at 4 °C, and analyse fresh plasma whenever possible. If immediate analysis cannot be performed, plasma samples may be stored at –80 °C for up to 2 months prior to analysis.
